# Methylene blue decreases brain mitochondrial ABAD and amyloid beta levels protecting mitochondrial functions in LPS-mouse model

**DOI:** 10.1186/1750-1326-8-S1-P1

**Published:** 2013-09-13

**Authors:** Reham M Abdel-Kader, Aya Zakaria, Nabila Hamdi

**Affiliations:** 1 Department of Pharmacology & Toxicology, German University in Cairo, Cairo, Egypt

## Background

Methylene blue (MB) is lately being proposed to be effective in treating Alzheimer’s disease (AD). Phase 2 clinical trials reported improvements in cognitive functions of AD patients after MB treatment. One of the main mechanisms of action that has been described for MB is inhibition of Tau aggregation [1]. Moreover, its antioxidant and mitochondrial protection have been previously described [2]. Only recently, a study using a triple transgenic AD mouse model has tested the mechanism of MB in vivo, showing improved cognition and reduced Aβ levels after MB treatment [3].

Recently, the mitochondrial enzyme Amyloid binding alcohol dehydrogenase (ABAD) has been shown to bind Aβ inducing mitochondrial dysfunction, providing a direct relation between Aβ and mitochondrial dysfunction occurring in AD. Previous studies have shown that inhibiting ABAD protects mitochondrial functions and prevented Aβ-induced toxicity [4][5]. Taking into consideration the mitochondrial protective effect of MB and the recent data suggesting its ability to reduce Aβ levels, our aim was to investigate the effect of MB on ABAD and mitochondrial function in an LPS mouse model that has been previously described to induce memory impairment, with Aβ accumulation in hippocampus and cerebral cortex [6].

## Materials and methods

LPS mouse model was used (I.P 250 µg/kg LPS for 7 consecutive days) and accumulation of Aβ was assessed by immunohistochemistry. To Test the effect of MB on this *in vivo* model, mice were treated for 4 days with MB (I.P 4 mg/kg). After sacrification of the mice, mitochondrial associated ROS and cell viability of brain cells were measured using dihydrorhodamine probe and MTT assay respectively. Moreover, ABAD and Aβ levels were determined by western blotting in the brains of the treated mice compared to the control group.

## Results

The LPS mouse model used in this study showed brain accumulation of Aβ, decreased cell viability and increased mitochondrial associated ROS levels compared to the vehicle group. MB treatment significantly increased cell viability and reduced the LPS-induced increase in the ROS level. Moreover, brain Aβ level of the MB treated group was significantly decreased compared to the untreated group. Most interestingly, MB treatment was able to reduce the high level of ABAD that was found in the LPS mouse model.

## Conclusions

Taken together our results showed that MB decreased both Aβ and ABAD levels, while protecting the mitochondria from oxidative stress, consequently improving brain cell viability. Based on the previously reported role of the interaction between ABAD and AB in inducing mitochondrial dysfunction, the current study suggests a novel mechanism of action of MB, linking its mitochondrial protective effects to its lowering effect of both AB and ABAD which may decrease their binding and the resultant mitochondrial stress.

